# Human Oral Epithelial Cells Impair Bacteria-Mediated Maturation of Dendritic Cells and Render T Cells Unresponsive to Stimulation

**DOI:** 10.3389/fimmu.2019.01434

**Published:** 2019-06-28

**Authors:** Magdalena Molero-Abraham, Jose L. Sanchez-Trincado, Marta Gomez-Perosanz, Alvaro Torres-Gomez, Jose Luis Subiza, Esther M. Lafuente, Pedro A. Reche

**Affiliations:** ^1^Department of Immunology, School of Medicine, Complutense University of Madrid, Madrid, Spain; ^2^Inmunotek SL, Madrid, Spain

**Keywords:** oral epithelial cells, dendritic cells, T cells, immunomodulation, bacteria

## Abstract

The oral mucosa is a first line of defense against pathogenic organisms and yet tolerates food antigens and resident bacteria. Mucosal epithelial cells are emerging as important regulators of innate and adaptive immune responses. However, the contribution of oral epithelial cells (OECs) determining oral immunity is understudied. Here, we evaluated the ability of H413 and TR146 cells, two OEC lines derived from human oral squamous cell carcinomas, and primary OECs to modulate immune responses to a cocktail of Gram^+^ and Gram^−^ bacteria known as MV130. OECs expressed CD40 constitutively and class II major histocompatibility complex (MHC II) molecules when stimulated with IFNγ, but not CD80 or CD86. Dendritic cells (DCs) treated with bacteria in co-culture with OECs did not fully mature, as judged by the expression of MHC II, CD80 and CD86, and barely released IL-12 and TNFα, compared to control DCs. Furthermore, in the presence of OECs, DCs were unable to stimulate allogenic naive CD4 T cells to produce IFNγ and TNFα. Similarly, OECs in culture with total CD4 T cells or Th1 cells stimulated with anti-CD3 and anti-CD28 antibodies abrogated CD25 and CD69 expression, T cell proliferation and the release of IFNγ and TNFα. The inhibition on T cell activation by OECs was cell-contact dependent, TGFβ independent and largely irreversible. Overall, this behavior of OECs is likely key to avoid immune system over-reaction against resident bacteria.

## Introduction

The oral mucosa constitutes a physical barrier between the inner and outer environment and is in charge of orchestrating first line immune responses against pathogenic intruders and harmful substances (1). Interestingly, oral epithelial cells are also in contact with commensal species and food antigens that are tolerated by the immune system. The oral mucosa differs from other mucosae in that it includes a stratified epithelium consisting of several layers of squamous epithelial cells piling up on top of the lamina propria with increasing degree of keratinization. Embedded in the oral epithelium there are abundant Langerhans cells (LCs) (CD11b^+^, CD11c^+^, CD1a^+^, CD207^+^), whereas the lamina propria contains myeloid DCs (mDCs) (CD11c^+^, CD209^+^) macrophages, plasma cells, B cells, and different subsets of T cells (1–3).

Epithelial cells in the mucosae can act as initial sensors of danger and have a decisive role in determining immune responses (4). In general, residents DCs in the mucosae orchestrate defense and tolerance mechanisms after the environmental context provided by the surrounding epithelial cells (4–6). For instance, intestinal epithelial cells (IECs) contribute to the generation of CD103^+^ DCs by releasing retinoic acid and TGFβ (7). Likewise, IECs promote non-inflammatory Th2 responses and Treg differentiation through the release of thymic stromal lymphopoietin (TSLP) (8, 9). This same cytokine is also produced by cryptic and squamous epithelial cells from inflamed tonsils and has been shown to trigger dendritic cell–mediated allergic inflammation (10).

Mucosal epithelial cells can also modulate T cell responses directly and act as non-professional antigen-presenting cells (4, 11–16). It has been shown that colonic, intestinal, and esophageal epithelial cells express MHC II and CD80/CD86 co-stimulatory molecules in response to IFNγ, working as non-professional antigen-presenting cells under pathological conditions (11–14). Colonic epithelial cells and human biliary epithelial cells reduce antigen-mediated activation of CD4 T cells by unknown mechanisms requiring cell contact (15, 16). Also human renal tubular epithelial cells suppress alloreactive T cell proliferation in a contact-dependent manner (17). In addition, kidney, airway, thymic and colonic epithelial cells constitutively express CD40 indicating that these cells can directly be influenced by T cell responses (15, 18–20).

Oral epithelial cells (OECs) are also likely to control oral immune responses but such a role is understudied. In this work, we used primary OECs isolated from the oral cavity of healthy volunteers and two human oral epithelial cell lines—H413 and TR146 cells, both from oral squamous cell carcinomas—with the aim to analyze their ability to condition the immune responses to bacterial stimuli. A cocktail of Gram^+^ and Gram^−^ bacteria known as MV130 was used (21). Besides containing bacteria genera and species well-represented in oral cavities (21, 22), MV130 has *in vivo* immunomodulary properties (23), and stimulates DCs and promotes T cell polarization (21) *in vitro*. We found that OECs hampered the maturation of DCs, reducing MHC II, CD80 and CD86 expression, and inhibited IL-12, IL-10, and TNFα secretion promoted by bacteria. Moreover, unlike DCs maturated with bacteria alone, DCs co-cultured with OECs were unable to induce the differentiation of IFNγ-producing CD4 T cells. Furthermore, OECs could directly impair the activation of CD4 T cells mediated by anti-CD3/CD28 antibodies, reducing CD25 and CD69 expression, T cell proliferation and IFNγ secretion in a contact-dependent manner, pointing to an inherent ability of OECs to suppress immune responses.

## Methods

### Oral Epithelial Cells (OECs) and Bacterial Cocktail

OEC lines H413 and TR146, derived from human oral squamous cell carcinomas, were acquired from the European Collection of Authenticated Cell Cultures (ECACC, Merck KGaA Darmstadt, Germany). Primary human OECs were obtained from voluntary healthy donors, as described by Michalczyk et al. (24), with slight modifications. All donors signed informed consent documents. We cultured H413 cells at 37°C and 5% CO_2_ in DMEM:HAMS F12 (1:1,vol/vol) (Gibco, NY, USA) supplemented with 0.5 μg/ml hydrocortisone sodium succinate (Merck KGaA, Darmstadt, Germany), TR146 cells in DMEM (Gibco, NY, USA), and primary OECs in RPMI 1640 (Gibco, NY, USA). In all cases, media were supplemented with 10% fetal bovine serum (FBS) (Gibco NY, USA), 100 U/ml penicillin, 100 μg/ml streptomycin, and 2 mM L-glutamine (Lonza, Walkersville, USA).

As bacterial stimuli, we used MV130 (Bactek®, Inmunotek S.L., Spain), a cocktail of inactivated whole cell bacteria (10^9^ bacteria/ml) containing *Staphylococcus aureus* (15%), *Staphylococcus epidermidis* (15%), *Streptococcus pneumoniae* (60%), *Klebisella pneumoniae* (4%), *Branhamella catarrhalis* (3%) and *Haemophilus influenzae* (3%).

### OECs Stimulation and Preparation of OEC-Conditioned Media

OECs were treated with 1,000 U/ml IFNγ (Immunotools) or with MV130 (10 bacteria:1 OEC) for 48 h on 96-well plates (2.5 × 10^4^ cells/well). To obtain the OEC-conditioned media, we collected OEC-culture supernatants, filtered them with a 0.22 μm diameter pore size filter and stored them at −20°C until further use. OECs and OEC-conditioned media (CM) treated with or without bacteria were subsequently used in cultures with DCs and/or T cells.

### Generation of DCs and Culture With OECs

DCs used in this study consisted in human monocyte-derived dendritic cells. Briefly, we first obtained peripheral blood mononuclear cells (PBMCs) from buffy coats from the regional blood transfusion center (Centro de Transfusion de la Comunidad de Madrid (Madrid, Spain). Donors previously signed the informed consent document for the use of organs and/or tissues for research purposes, following the legislation corresponding to the Royal Decree-Law 1088/2005 of September 16 (reference number: BOE-A-2005-15514). PBMCs were isolated by a density gradient on Ficoll-Paque™ PLUS (Amershan) and subsequently purified CD14^+^ monocytes by positive selection using magnetic beads coupled with an anti-CD14 antibody (Miltenyi Biotec). CD14^+^ cells were plated on 24-well plates (1.5 × 10^6^ cells/well) and incubated for 5 days in complete RPMI medium supplemented with 800 U/ml granulocyte-macrophage colony-stimulating factor (GM-CSF) and 400 U/ml IL-4 (Immunotools). The resulting immature DCs were used in experiments in which they were incubated on 96-well plates (10^5^ cells/well) for 48 h with OECs (4 DC:1 OEC) pretreated with or without stimuli (MV130) (10 bacteria:1 OEC).

### DC Stimulation of CD4 T Cells in Culture With OECs

DCs were used to stimulate allogenic naive CD4 T cells purified from PBMCs using antibody-coupled magnetic beads (human CD4 T cell and naive CD4 T cell isolation kits, Miltenyi Biotec). CD4 T cells were plated on 96-well plates in complete RPMI medium (2 × 10^5^ cells/well) including: DCs (1 × 10^5^ cells/well) alone (controls) previously treated with or without MV130 for 48 h (10 bacteria:1 T cell), DCs and OECs treated or untreated with MV130 (8 T cell:4 DC:1 OEC). Cell cultures were then incubated at 37°C and 5% CO2 for 6 days, adding 10 ng/ml IL-2 (Immunotools) every 2 days.

### CD3/CD28-Mediated Activation of CD4 T Cells in Culture With OECs or OEC-Conditioned Media

We carried out antibody-stimulations on naive, total, and Th1 CD4 T cells using anti-CD3/CD28 antibodies. Total and naive CD4 T cells were isolated from PBMCs as indicated previously. Th1 cells were generated from naive T cells by incubating them on 96-well plates (2 × 10^5^ cells/well) with beads coupled with anti-CD3/CD28 antibodies (Dynabeads® Human T-activator CD3/CD28, ThermoFisher Scientific) in complete RPMI medium supplemented with 10 ng/ml IL-12 (Immunotools) and 5 μg/ml anti-IL-4 antibody (Sigma-Merck KGaA, Darmstadt, Germany). Subsequently, beads were removed following the manufacturer's instructions and Th1 cells were left resting for 6 h. Naive, total or Th1 CD4 T cells were stimulated by incubating them on 96-well plates (2 × 10^5^ cells/well) in complete RPMI with Dynabeads® Human T-activator for either 48 h for Th1 and total CD4 T cells or 6 days for naive T cells. For each CD4 T cell subset, antibody-mediated stimulations were performed under three different experimental contexts: CD4 T cells alone (controls), with or without MV130 (10 bacteria:1 T cell); CD4 T cells in co-culture with OECs previously treated for 48 h with or without MV130 (8 T cell:1 OEC) and CD4 T cells with OEC-conditioned media, obtained as indicated before. In some experiments, CD4 T cells were re-activated with Dynabeads® Human T-activator after OEC co-culture.

### CD4 T Cell Proliferation and Apoptosis Assays

Anti-CD3/CD28 activated CD4 T cells were labeled with Carboxyfluorescein Diacetate Succinimidyl Ester (CFSE) (ThermoFisher) following the manufacturer's instructions and plated on 96-well cell-culture plates (2 × 10^5^ cells/well) in complete RPMI media with or without OECs (8 T cell:1 OEC), that were previously treated for 48 h with or without MV130. Plates were incubated at 37°C and 5% CO2 for 7 days. Proliferation of CFSE-labeled CD4 T cells was monitored by flow cytometry (BD FACSCalibur). For apoptosis assays, anti-CD3/CD28 activated CD4 T cells in complete RPMI media on 96-well plates (2 × 10^5^ cells/well) were incubated alone or with OECs (8 T cell:1 OEC) previously treated with or without MV130. After 48 h of culture at 37°C and 5% CO2, cell necrosis/apoptosis was assessed by flow cytometry (BD FACSCalibur) using Annexin V Apoptosis Detection Kit with 7-AAD (Biolegend).

### Analysis of Cell Surface Markers and Cytokine Production

Cell surface markers expression on OECs, DCs, and CD4 T cells was analyzed by flow cytometry using the following antibodies and kits: anti-CD80 (L307.4), anti-CD83 (HB15e) and anti-CD86 (FUN-1), all from BD Pharmingen; anti-HLA-ABC (REA230), anti-HLA-DR (AC122), anti-CD83 (HB15), anti-CD40 (HB14) and anti-Cd11c (MJ4-27G12), all from Miltenyi-Biotech; anti-CD25 (BC96) and anti-CD69 (FN50) (eBioscience) and FITC Annexin V Apoptosis Detection Kit with 7-AAD (Biolegend), following the manufacturer's instructions. Briefly, cells were washed with 0.5% BSA/2 mM EDTA in PBS (staining buffer), Fc receptors blocked with human IgG serum (200 μg/ml)(Sigma-Merck) for 30 min at RT, and stained with the fluorescence-labeled antibodies. Cell markers expression was measured by flow cytometry (BD FACSCalibur) and data analysis was performed using FlowJo software (Tree Star).

IFNγ, TNFα, TGFβ, IL-1β, IL-2, IL-4, IL-6 and IL-10 (Invitrogen), and IL-8 and IL-12 (Inmunotools) were quantified in cell-free supernatants using specific solid phase sandwich ELISA cytokine kits following the manufacturer's instructions. The resulting color was measured (BioTek ELx800 Absorbance Microplate Reader).

### TGFβ Blocking Assay

We incubated H413 OECs on 96-well plates (2.5 × 10^5^ cells/well) in complete RPMI in the presence or absence of MV130 for 48 h. Subsequently, we added increasing concentrations (20 ng/ml, 300 ng/ml or 2.5 μg/ml) of a blocking anti-TGFβ antibody (Anti-hTGFβ-IgA, Invivogen) and after a 4 h incubation we added total CD4 T cells (8 T cell:1 OEC), previously activated with anti-CD3/CD28 beads (human T-activator CD3/CD28). We incubated the plates for 48 h, and detected IFNγ and TNFα levels by ELISA kits (Invitrogen).

### Statistical Analysis

Data are expressed as the mean ± standard error of the mean. We applied two-tailed Student's *t*-test for independent samples to assess statistical significance between means (Welch's test), assuming normality and heteroscedastic variances. *P* < 0.05 was considered significant. Statistic calculations were performed on Microsoft Excel (2016).

## Results

### Cytokine Secretion and Expression of HLA-DR and Co-stimulatory Molecules by OECs

**Figure 1:**
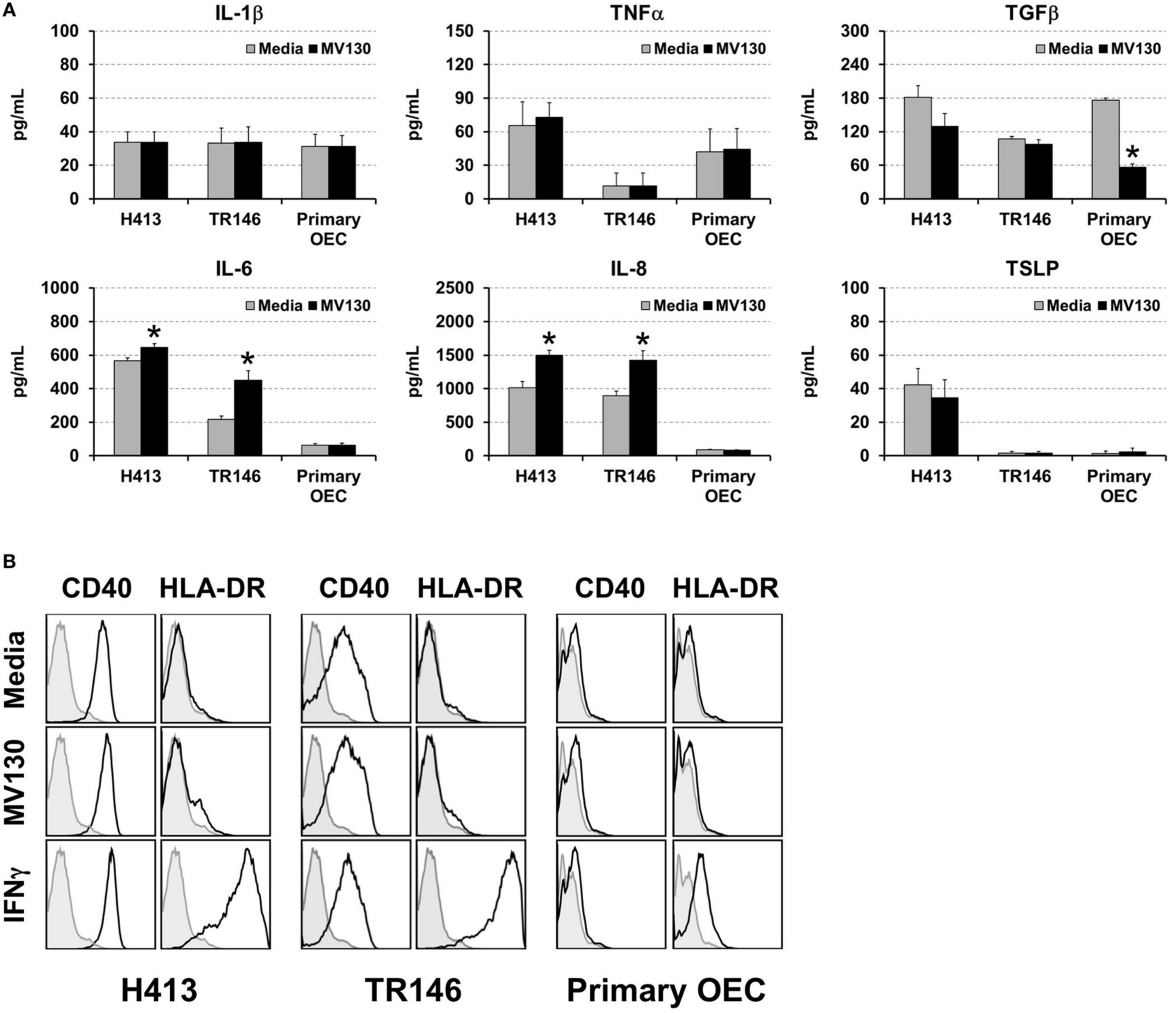
Cytokine secretion and surface markers expression in OECs. **(A)** Figure depicts the levels of various cytokines determined by ELISA on cell culture supernatants obtained from H413 and TR146 OEC lines and primary OECs incubated with media (gray bars) or the bacterial cocktail MV130 (black bars). Statistically significant differences (*p* < 0.05) between media and MV130 conditions are represented by “*”. Data were obtained from a total of seven independent experiments and we plotted mean values with error bars corresponding to standard errors of the mean (SEM). **(B)** Figure shows surface expression of CD40 and HLA-DR in H413, TR146 and primary OECs cultured with media, MV130 or IFNγ for 48 h, as determined by flow cytometry (black profiles). Isotype specific antibodies were used as negative control (gray profiles). Result shows MFI of a representative experiment out of three independent assays.

We analyzed cytokine secretion in two human oral epithelial cell (OEC) lines derived from squamous cell carcinomas (H413 and TR146) and in primary OECs, obtained from the oral cavity of healthy volunteers. OECs were treated or untreated with a cocktail of whole cell heat-inactivated Gram^+^ and Gram^−^ bacteria (MV130) (10:1, bacteria:OEC ratio). After 48 h, the levels of IL-1β, TNFα, TGFβ, IL-6, IL-8, and TSLP were determined in culture supernatants. As shown in Figure 1A, H413 and TR146 cell lines, but not primary OECs, constitutively secreted IL-6 and IL-8, and this secretion increased slightly under bacterial stimuli, whereas TNFα and IL-1β secretion remained unchanged before and after bacterial exposure. TGFβ was secreted by all cell types at similar levels and decreased in primary OECs after bacterial contact. TSLP was only detected in H413 cultures and its production was not modified by bacterial stimulation.

Different studies have shown that under certain inflammatory conditions epithelial cells can express HLA-DR and co-stimulatory markers (25). To investigate this issue, H413, TR146 and primary OECs were treated with MV130 or with IFNγ, a well-known HLA-DR inducer, as control. Subsequently, cells were analyzed by flow cytometry for CD80, CD86, CD83, CD40 and HLA-DR expression. OECs did not express CD80, CD86 or CD83 under any of the assayed conditions (data not shown). CD40 was expressed at high levels in H413 and TR146 cells but not in primary OECs, being unaltered by bacteria or IFNγ stimulation (Figure 1B). HLA-DR expression was substantially induced on H413 and TR146 cell lines and in a lower extent in primary OECs when treated with IFNγ, but invariable when treated with MV130. Overall, these results indicate that OECs were poor responders to the assayed stimuli, and only in the presence of IFNγ, HLA-DR can be upregulated.

### Effect of OECs on Dendritic Cell Maturation and Cytokine Secretion

**Figure 2:**
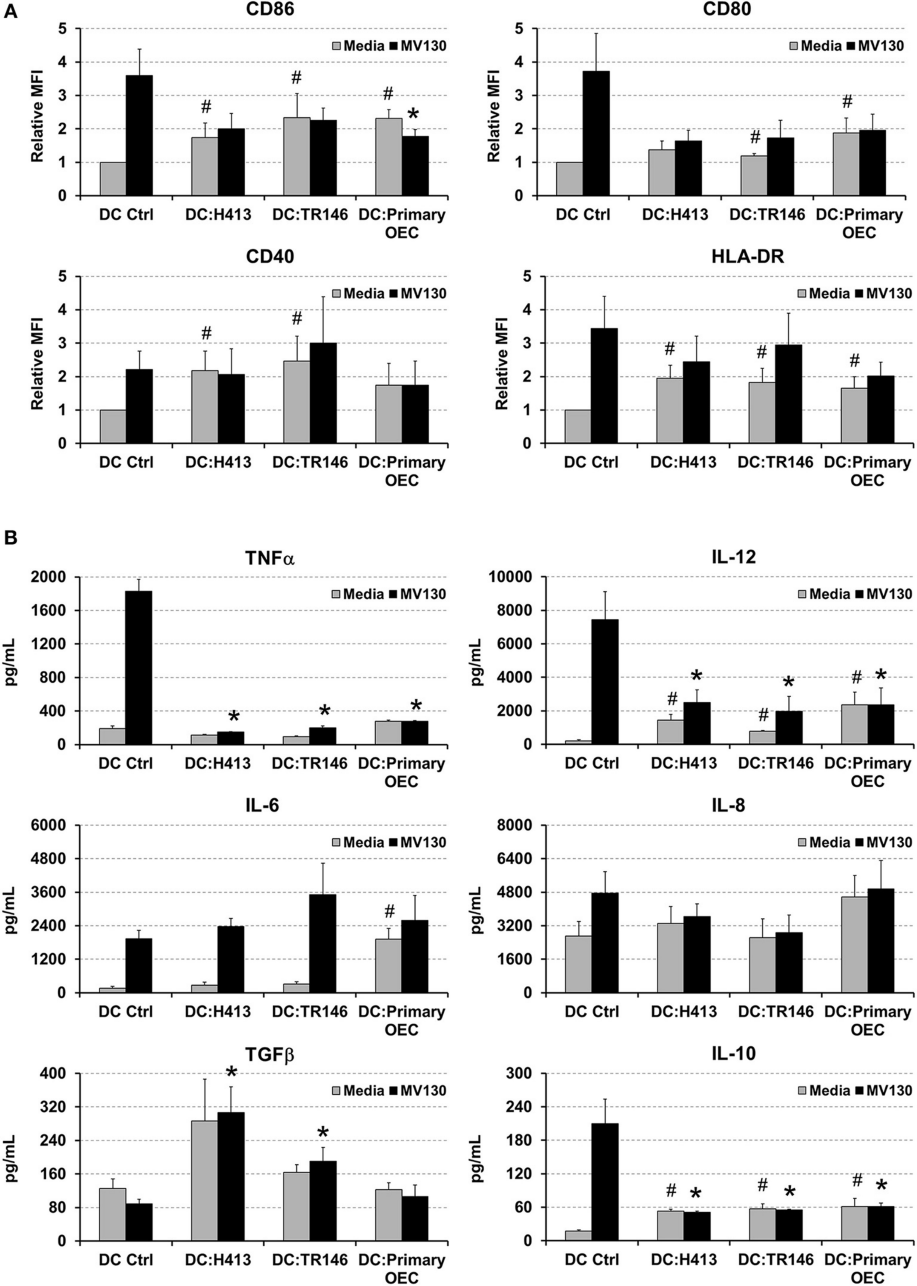
Effect of OECs on DC maturation and cytokine expression. **(A)** Expression of CD86, CD80, CD40 and HLA-DR in monocyte-derived DCs cultured alone (DC Ctrl) or in co-culture with OECs during 48 h (DC:H413; DC:TR146; DC: primary OECs). Black bars represent cells incubated with MV130 and gray bars represent cells in media. DCs are gated based on CD11c staining. **(B)** Cytokine secretion by DCs cultured with OECs. Figure shows the levels of TNFα, IL-12, IL-6, IL-8, IL-10 and TGFβ (pg/ml) determined by ELISA in supernatants of DCs cultured alone (DC Ctrl) or in co-culture with OECs during 48 h (DC:H413; DC:TR146; DC: primary OECs). Black bars represent cells incubated with MV130 and gray bars represent cells with media. Statistically significant differences (*p* < 0.05) between the different DC conditions and DC Ctrl were noted as “^#^” for media or as “*” for MV130. Data were obtained from a total of four independent experiments and we plotted mean values with error bars corresponding to SEM.

Since a crosstalk between epithelial cells and DCs has been described (6, 10), we addressed whether OECs could influence the maturation and cytokine production of DCs. These experiments were performed with OECs that were previously treated with or without bacteria and then co-cultured with monocyte-derived DCs, as described in Methods. DCs were also cultured alone with or without the same bacterial cocktail as control. As observed in Figure 2A, MV130 increased the expression of CD86, CD80, CD40, and HLA-DR on DCs cultured alone. In contrast, the increment in expression for these molecules (except for CD40) was greatly reduced when DCs were co-cultured with OECs (DC:OEC) (Figure 2A). Note that bacteria were also present in this co-culture. Interestingly, in the absence of bacteria, OEC-instructed DCs increased their expression of CD86, CD40 and HLA-DR compared to DCs alone (Figure 2A).

Figure 2B shows cytokine production in 48 h culture supernatants in the same co-culture system as above. As expected, control DCs treated with MV130 produced high levels of TNFα, IL-12, IL-6 and IL-10. In contrast, DCs co-cultured with OECs and MV130 produced lower levels of TNFα, IL-12 or IL-10, while TGFβ and IL-6 levels increased or remain unmodified. Overall, these results indicated that OECs, independently of bacterial stimulation, may promote DC maturation but holding these cells in a semi-maturation state, as judged by the reduced expression of MHC II and co-stimulatory molecules as well as the impaired production of IL-12 and TNFα after challenging with strong bacterial stimuli.

### Effect of OEC Modulation Over DC-Mediated T Cell Polarization

**Figure 3:**
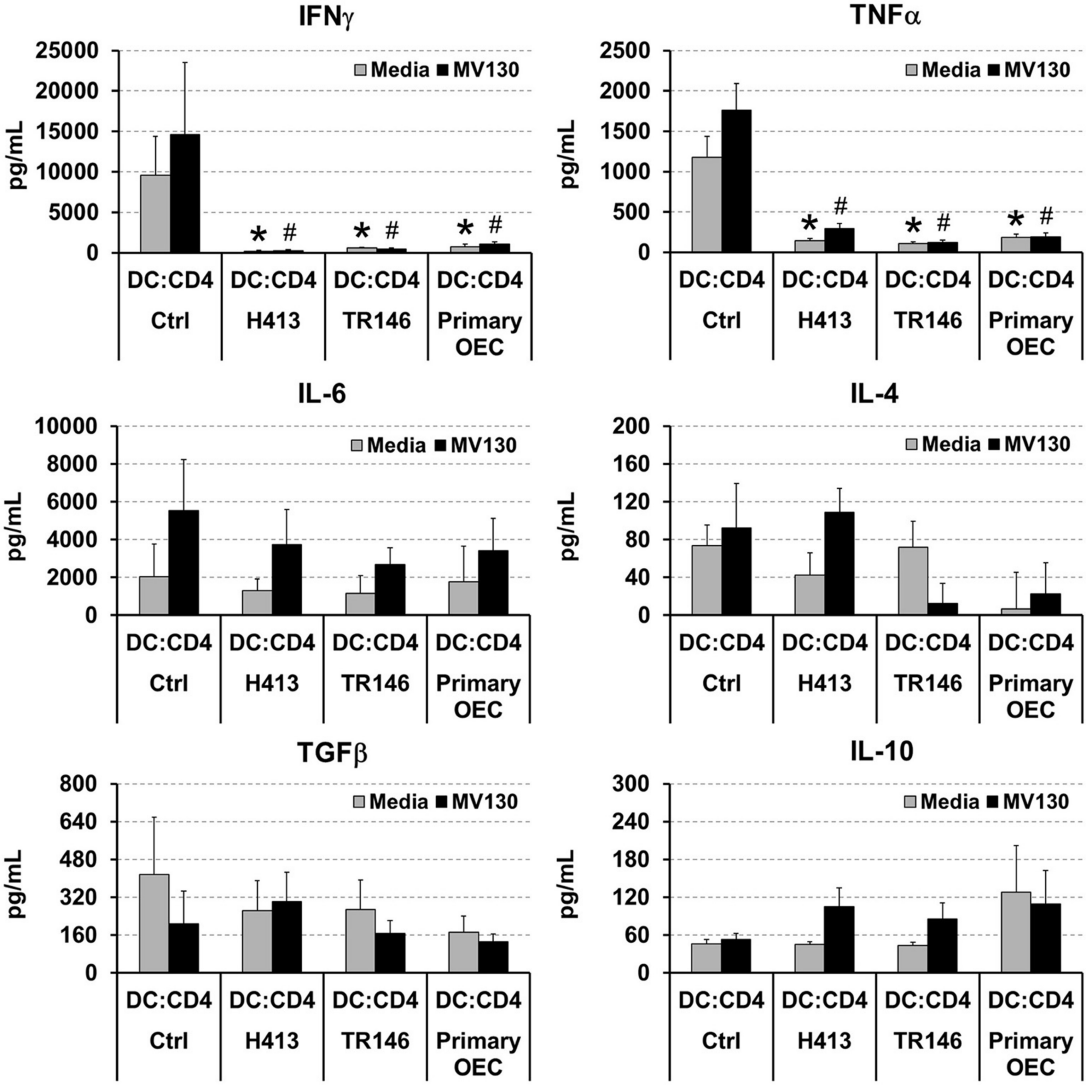
Analysis of cytokines in DC:T cell cultures under OEC conditioning. Figure depicts the levels of IFNγ, TNFα, IL-6, IL-4, TGFβ and IL-10 detected by ELISA in cultures of naive CD4 T cells activated by allogenic DCs (DC:CD4) alone (controls) or in co-culture with H413, TR146, or primary OECs (DC:OEC) with media (gray bars) or MV130 (black bars). Statistically significant differences (*p* < 0.05) between the different DC:CD4 conditions and DC:CD4 Ctrl were noted as “^#^” for media or as “*” for MV130. Data were obtained from a total of three independent experiments and we plotted mean values with error bars corresponding to SEM.

DCs link innate immunity and adaptive immunity by driving the activation and differentiation of naive T cells. Thereby, we evaluated the ability of DCs to activate and polarize naive T cells under OEC conditioning with or without bacteria. To that end, allogenic naive CD4 T cells were cultured for 6 days with DCs alone (controls) or DCs co-cultured with OECs, with or without MV130 as described in Methods, and IFNγ, TNFα, IL-4, IL-6, TGFβ and IL-10 were measured in the resulting culture supernatants. As shown in Figure 3, control cultures containing CD4 T cells and allogenic DCs produced detectable amounts of all these cytokines that remained stable or increased in the presence of bacteria, with the exception of TGFβ, that decreased under bacterial exposure. Interestingly, IFNγ and TNFα were barely detectable in DC:CD4 T cell cultures containing OECs, regardless of the presence of bacteria (Figure 3), in contrast to control DC:T cell that expressed large amounts of these two cytokines (Figure 3). Similar results were observed in the absence of DCs, when CD4 T cells were activated through CD3/CD28 stimulation in the presence of OECs. However, this inhibitory effect was not observed when activated CD4 T cells were incubated only with OECs culture media, hereafter OEC-conditioned media (CD4:CM), pointing to a direct effect of OECs on T cells (Supplemental Figure 1).

**Figure 4:**
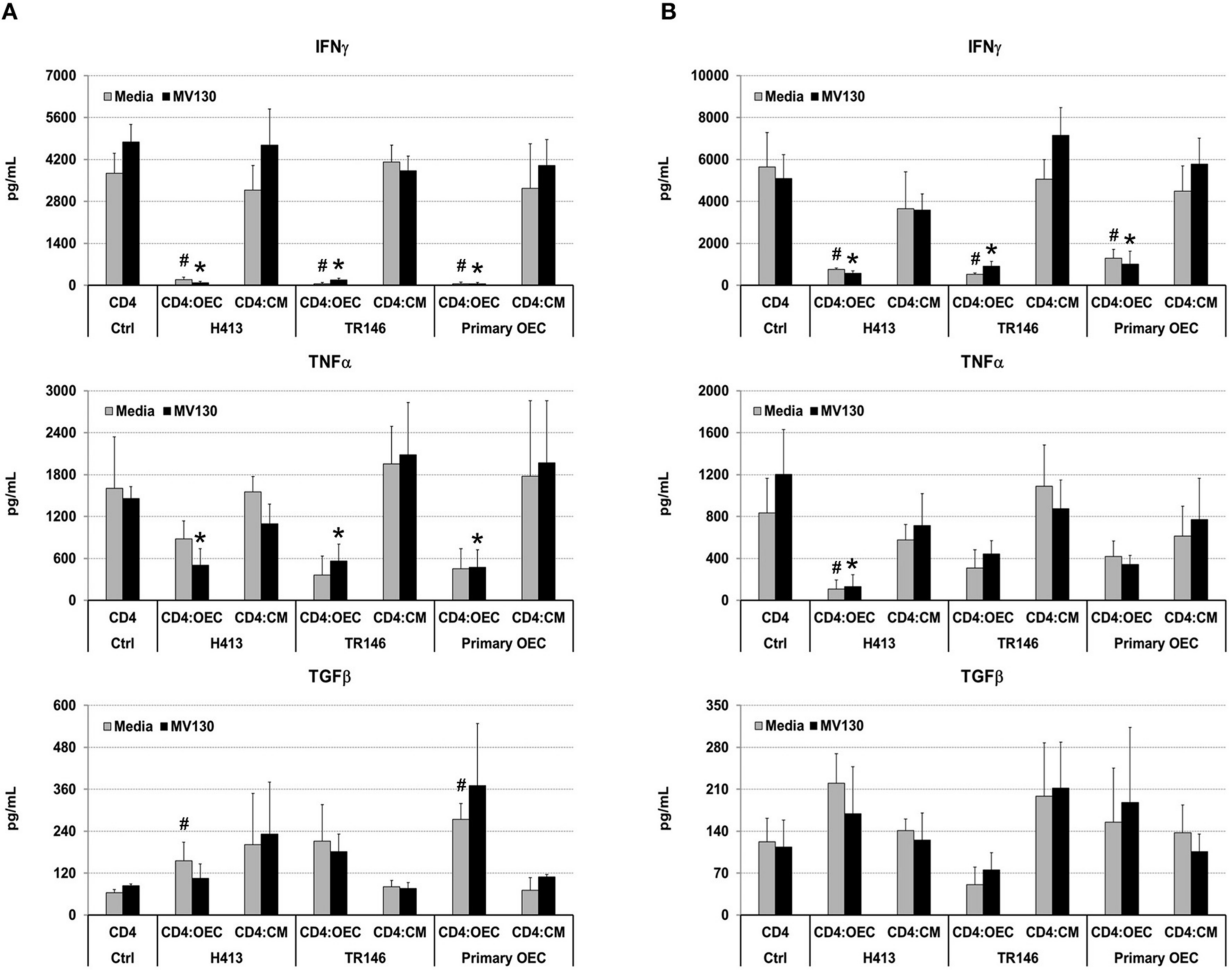
OECs alter the cytokine levels produced by CD4 T cells. Levels of IFNγ, TNFα and TGFβ determined by ELISA on cultures consisting of CD3/CD28 activated total CD4 T cells **(A)** or Th1 cells **(B)** alone (CD4 Ctrl), in the presence of OEC (CD4:OEC) or their conditioned media (CD4:CM). The experiments were carried out using H413, TR146 cell lines and primary OECs. Media and MV130 conditions are represented by gray and black bars, respectively. Statistically significant differences (*p* < 0.05) between the different CD4 T cells conditions and CD4 T cells Ctrl were noted as “^#^” for media or as “*” for MV130. Data were obtained from a total of three independent experiments and we plotted mean values with error bars corresponding to SEM.

Next, we analyzed if OECs, with or without bacterial stimuli, could also suppress IFNγ production by T cells that have already undergone differentiation. To that end, we activated total CD4 T cells and Th1 cells (differentiated *in vitro*, details in Methods) with anti-CD3/CD28 antibodies, alone (CD4 control) or in the presence of OECs (CD4:OEC) or OEC-conditioned media (CD4:CM), including conditions with and without MV130. After 48 h, we assessed CD4 T cell responses by determining IFNγ, TNFα, and TGFβ in cell culture supernatants. As shown in Figure 4, CD3/CD28 stimulation of total CD4 T cells or Th1 cells triggered the secretion of vast amounts of IFNγ that were readily suppressed by the presence of OECs but not by their conditioned media. A similar suppression was also observed with respect to the production of TNFα, albeit to a lesser extent. By contrast, TGFβ was in general increased in CD4:OEC co-cultures especially when using primary OECs. Again, the presence or absence of bacteria did not alter these results (Figure 4).

A summary of the most representative cytokines (IL-6, IL-10, IL-12, IFNγ, TNFα and TFGβ) produced in the presence or absence of MV130 during different culture conditions, is shown in Table 1.

**Table 1:** Summary of the cytokines produced by OEC/DC/CD4-culture systems in the presence or absence of MV130.

	**Culture conditions**		**Assayed cytokines^a^**					
	**OEC**	**MV130**	**IL-6**	**IL-10**	**IL-12**	**IFNγ**	**TNFα**	**TGFβ**
–	H413	**–**	567 ± 17	n/a	n/a	n/a	65 ± 21	181 ± 20
		**+**	645 ± 22	n/a	n/a	n/a	72 ± 13	129 ± 23
	TR146	**–**	217 ± 19	n/a	n/a	n/a	11 ± 11	107 ± 4
		**+**	449 ± 56	n/a	n/a	n/a	11 ± 11	97 ± 7
	PRIM OEC	**–**	62 ± 11	n/a	n/a	n/a	42 ± 20	176 ± 3
		**+**	64 ± 10	n/a	n/a	n/a	44 ± 18	56 ± 5
DC	–	**–**	164 ± 64	17 ± 2	211 ± 55	n/a	188 ± 31	125 ± 22
		**+**	1,927 ± 308	210 ± 43	7,450 ± 1,658	n/a	1,829 ± 138	88 ± 10
	H413	**–**	275 ± 105	53 ± 3	1,446 ± 330	n/a	111 ± 10	286 ± 100
		**+**	2,372 ± 281	51 ± 2	2,491 ± 754	n/a	146 ± 8	306 ± 61
	TR146	**–**	318 ± 71	57 ± 9	784 ± 43	n/a	95 ± 9	163 ± 18
		**+**	3,509 ± 1,130	55 ± 1	1,962 ± 903	n/a	199 ± 24	190 ± 33
	PRIM OEC	**–**	1,920 ± 379	61 ± 14	2,348 ± 762	n/a	276 ± 15	123 ± 16
		**+**	2,589 ± 895	61 ± 6	2,349 ± 1,021	n/a	276 ± 8	106 ± 27
DC:CD4	–	**–**	2,031 ± 1,742	45 ± 7	n/a	9,605 ± 4,800	1,177 ± 258	416 ± 242
		**+**	5,527 ± 2,716	53 ± 9	n/a	1,4611 ± 8,883	1,757 ± 335	206 ± 138
	H413	**–**	1,301 ± 623	45 ± 4	n/a	200 ± 97	140 ± 27	264 ± 126
		**+**	3,753 ± 1,839	104 ± 30	n/a	267 ± 92	293 ± 64	301 ± 124
	TR146	**–**	1,164 ± 922	43 ± 5	n/a	562 ± 87	112 ± 15	269 ± 122
		**+**	2,678 ± 891	85 ± 25	n/a	485 ± 127	123 ± 23	168 ± 53
	PRIM OEC	**–**	1,782 ± 1,874	127 ± 74	n/a	745 ± 303	182 ± 42	172 ± 67
		**+**	3,423 ± 1,689	109 ± 52	n/a	1,049 ± 306	193 ± 43	133 ± 31
CD4	–	**–**	n/a	55 ± 16	n/a	11,941 ± 1,865	3,246 ± 451	116 ± 30
		**+**	n/a	58 ± 18	n/a	23,698 ± 4,470	4,815 ± 1,047	202 ± 40
	H413	**–**	n/a	42 ± 3	n/a	950 ± 238	226 ± 156	545 ± 100
		**+**	n/a	42 ± 2	n/a	869 ± 778	205 ± 129	536 ± 107
	TR146	**–**	n/a	43 ± 3	n/a	844 ± 242	374 ± 81	464 ± 66
		**+**	n/a	48 ± 6	n/a	900 ± 328	377 ± 110	315 ± 40
	PRIM OEC	**–**	n/a	35 ± 10	n/a	6,432 ± 2,309	3,870 ± 400	148 ± 41
		**+**	n/a	40 ± 1	n/a	7,613 ± 2,793	4,026 ± 359	59 ± 7
	H413-CM	**–**	n/a	59 ± 15	n/a	12,573 ± 1,882	1,909 ± 264	122 ± 24
		**+**	n/a	45 ± 4	n/a	12,388 ± 2,095	1,428 ± 207	106 ± 29
	TR146	–	n/a	46 ± 4	n/a	15,354 ± 3,335	2,586 ± 397	267 ± 51
		+	n/a	45 ± 4	n/a	14,295 ± 1,501	2,952 ± 124	171 ± 34
	PRIM OEC-CM	**–**	n/a	56 ± 17	n/a	11,183 ± 1,263	3,566 ± 385	69 ± 11
		**+**	n/a	43 ± 4	n/a	17,472 ± 5,579	3,547 ± 343	56 ± 7

a *Data are represented as mean ± SEM. n/a: not available. Units in pg/ml*.

### Analysis of the OEC-Mediated Suppression of CD4 T Cell Responses

**Figure 5:**
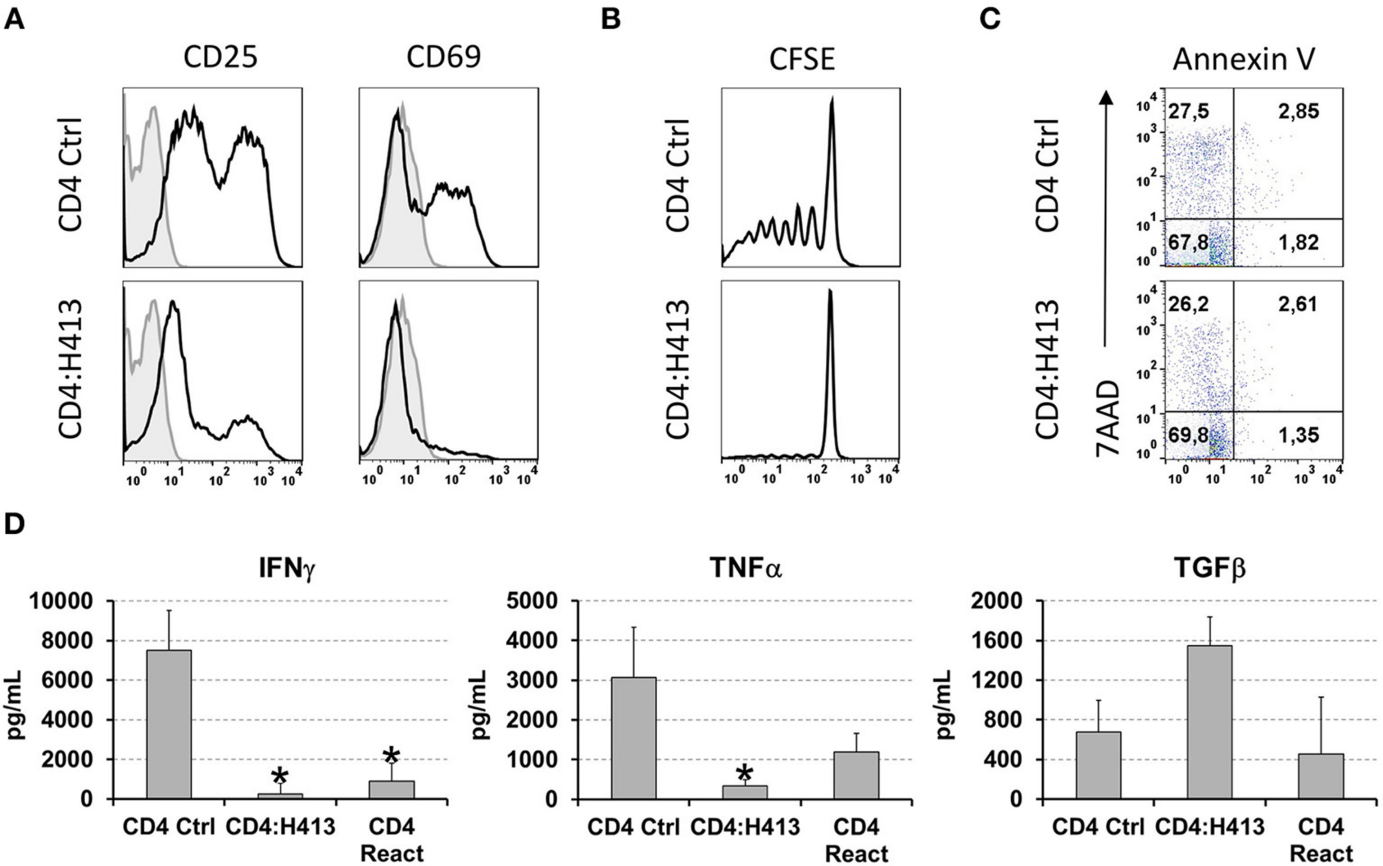
OECs suppressed the activation and proliferation of activated CD4 T cells. **(A)** Expression of CD25 and CD69 activation markers on CD3/CD28 stimulated CD4 T cells cultured alone or with H413 cells. **(B)** CFSE staining and **(C)** annexin V and 7-AAD staining of activated CD4 T cells alone (CD4) or in culture with OEC (CD4:H413). **(D)** IFNγ, TNFα, and TFGβ on supernatants of CD3/CD28 stimulated CD4 T cell cultures consisting of: control CD4 T cells (CD4 Ctrl); CD4 T cells cultured with H413 cells (CD4:H413) and the same CD4 T cells isolated from H413 co-cultures and activated again with anti-CD3/CD28 antibodies (CD4 React). Statistically significant differences (*p* < 0.05) between the different CD4 T cells conditions and CD4 T cells Ctrl were noted with “*”. Data were obtained from a total of three independent experiments and we plotted mean values with error bars corresponding to SEM.

To gain insight into the possible mechanism(s) by which OECs suppressed CD4 T cells IFNγ and TNFα production, different experiments were carried out using CD3/CD28-stimulated CD4 T cells in co-culture with H413 cells, as representative OEC and without the inclusion of bacteria. As shown in a representative experiment (Figure 5A), CD4 T cells expressed high levels of the T cell activation markers CD25 and CD69 upon stimulation, as expected. However, when H413 cells were present, CD25 was greatly decreased, and CD69 expression was virtually abolished suggesting a defect in T cell activation. Because of the lower expression of CD25 in co-culture with OECs (H413), the effect of these cells on T cell proliferation and viability was assessed. As shown in Figure 5B, CD3/CD28-stimulated CD4 T cells were unable to proliferate in the presence of H413 epithelial cells, without showing any increase in apoptosis or necrosis (Figure 5C).

Next, we asked whether OEC-conditioned CD4 T cells could respond to a new stimulation once they were re-isolated from OEC co-cultures. Thus, CD4 T cells were cultured alone or with H413 cells and 48 h later IFNγ, TNFα and TGFβ levels were measured. Latter, CD4 T cells were recovered from the culture, rested for 6 h and stimulated again (reactivated) with anti-CD3/CD28 antibodies. After 48 h, IFNγ, TNFα and TGFβ were analyzed in the supernatants. As shown in Figure 5D, reactivated CD4 T cells produced less amounts of IFNγ and TNFα than control activated CD4 T cells. In contrast, TFGβ production was similar to that of control CD4 T cells.

**Figure 6:**
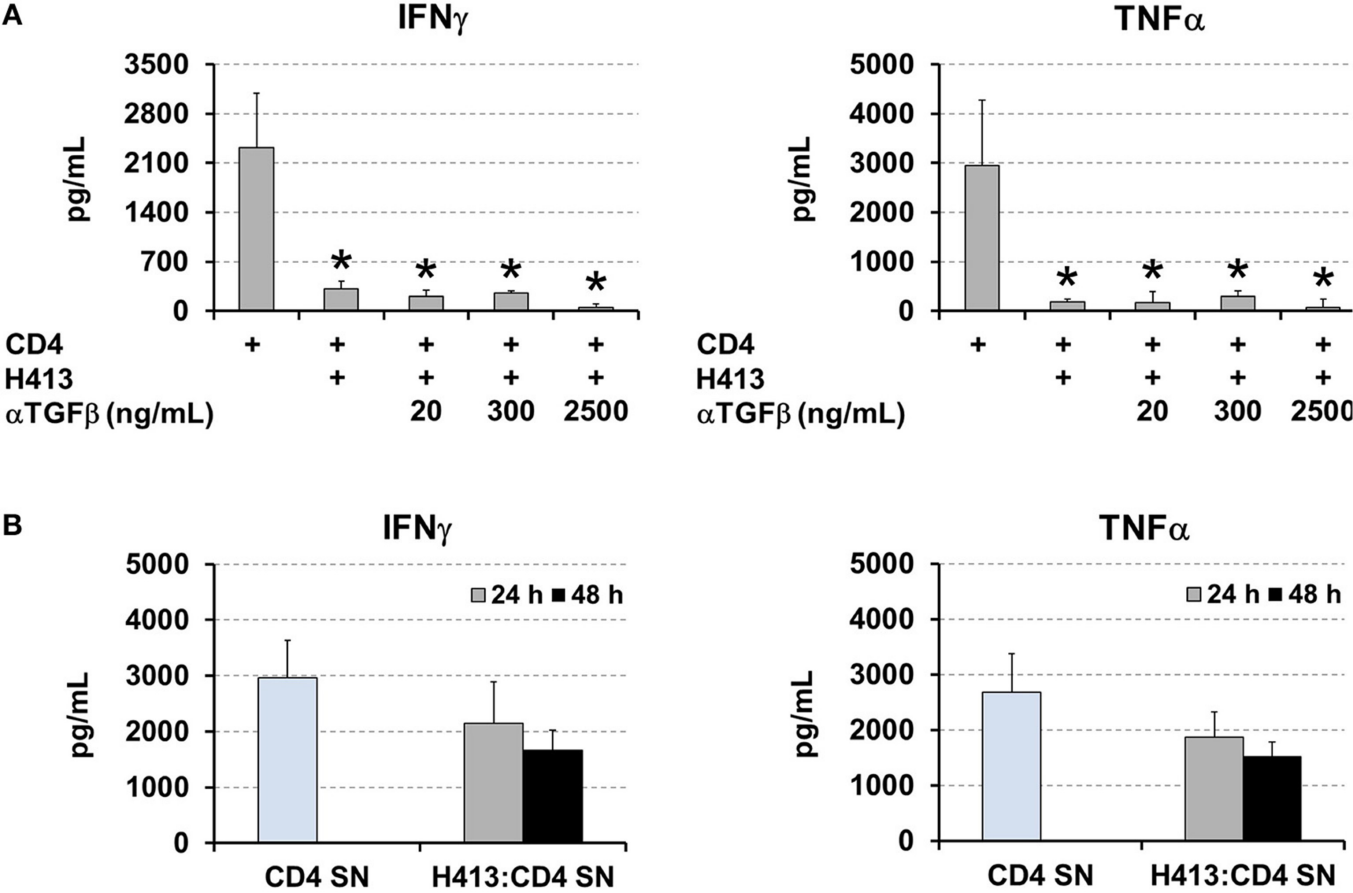
OEC-conditioning is not determined by TGFβ or cytokine consumption. **(A)** IFNγ and TNFα levels measured in co-cultures of H413 and CD3/CD28 stimulated CD4 T cells containing different concentrations of anti-TGFβ blocking antibody (ng/ml). Activated CD4 T cells alone were used as control. **(B)** Supernatants of CD3/CD28 activated CD4 T cells (CD4 SN) were assayed for IFNγ and TNFα prior to and after 24 and 48 h (gray and black bars, respectively) of being added to H413 cell cultures (H413: CD4 SN). Statistically significant differences (*p* < 0.05) shown in panel A between different CD4 T cell cultures and control CD4 T cells (left column) are noted as “*”. Data were obtained from a total of three independent experiments and we plotted mean values with error bars corresponding to SEM.

TGFβ production was active on OECs cultures (Figure 1A) and it was maintained and even increased, in the above various co-culture systems (Figures 2–4). As this cytokine may inhibit T cell proliferation and Th1 differentiation (26) we examined its role in the above inhibition of IFNγ and TNFα production. Thus, the same co-culture system as above (CD3/CD28-stimulated CD4 T cells and H413 epithelial cells) was performed in the presence of increasing amounts of anti-TGFβ blocking antibodies (Figure 6A). As shown, no effect was found regarding an increase in IFNγ and TNFα production as consequence of TGFβ blocking. Since epithelial cells can respond to IFNγ and TNFα (27), we investigated whether OECs might be depleting both cytokines from the media. To that end, CD3/CD28-stimulated CD4 T cells were cultured for 24 h and their supernatant added to H413 cells. After 24 and 48 h IFNγ and TNFα levels were measured and compared with the original supernatant, kept as control. The results shown in Figure 6B indicate that H413 cells could only deplete ~50% of IFNγ and TNFα produced by activated CD4 T cells.

## Discussion

The relevance of mucosal epithelial cells in the regulation of immune responses, particularly toward the maintenance of tolerance to foreign non-pathogenic antigens, has been highlighted in different studies generally involving the gut mucosa (28) and respiratory mucosa in the context of asthma development [reviewed in (29)]. However, the immunological role of epithelial cells in other mucosa, including the oral mucosae, remains largely understudied. The oral mucosa is a site of intense immunological activity, which also serves as a niche for numerous commensal bacteria. In the present work, we have analyzed the response of oral epithelial cells (OECs) to a cocktail of bacteria (MV130) and their ability to condition the activity of DCs and CD4 T cells. As OECs we used keratinocytes isolated from the inner cheek of healthy volunteers and H413 and TR146 cells, derived from human oral squamous cell carcinomas. H413 and TR146 cells maintain the epithelial characteristics of the parental cells and have been used as a model of oral epithelial cells in diverse studies (30, 31). MV130 is a mixture of inactivated whole cell Gram^+^ and Gram^−^ bacteria frequently found in the upper respiratory mucosa (32). We used MV130 for being a known trigger of DC maturation and T cell polarization (21). However, it is worth noting that the genus *Streptococci, Haemophilus*, and *Neisseria*, as well as *Stapphylococcus aureus*, all of them in MV130, are well represented in the commensal flora of the oral cavity (22). Moreover, *Streptococcus pneumoniae* in MV130 is closely related to the highly prevalent *S. mitis* and *S. oralis* within the oral flora, sharing more than 99% DNA sequence homology to each others (33).

**Figure 7:**
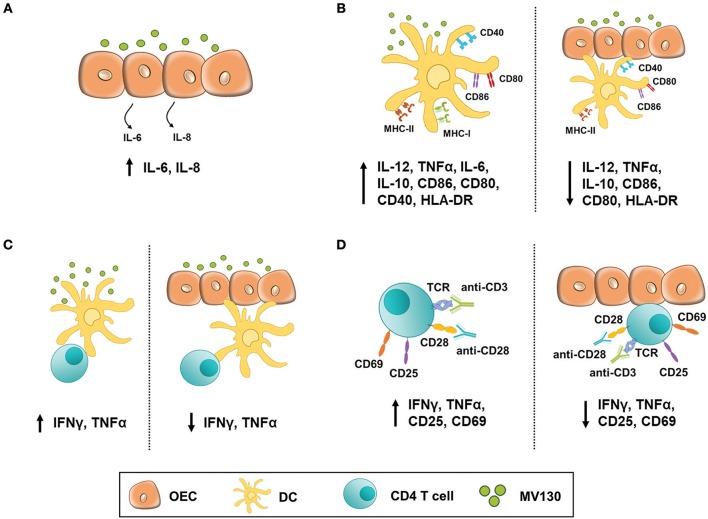
OEC-mediated immunomodulation under bacterial stimulation. Figure illustrates the experimental settings used in this work and the most relevant findings. **(A)** OECs response to MV130 stimulation. H413 and TR146 cells but not primary OECs constitutively released IL-6 and IL-8. These cytokines level increased with MV130 stimulation. **(B)** DCs did not fully mature in the presence of OECs. DC stimulated with MV130 expressed CD80, CD86 and HLA-DR, and released IL-12, IL-10 and TNFα (left), while in co-culture with OECs (right) showed decreased expression of HLA-DR, CD80 and CD86, and decreased release of IL-12, IL-10, and TNFα. **(C)** DCs failed to stimulate allogenic CD4 T cells in the presence of OECs. CD4 T cells stimulated with allogenic DCs previously treated with MV130 (left) released IFNγ and TNFα but did not when in co-culture with OEC (right). **(D)** CD4 T cell activation was compromised in the presence of OECs. CD3/CD28-activated CD4 T cells expressed CD25 and CD69 and released IFNγ and TNFα (left) unlike their counterparts in co-culture with OECs (right).

The experimental settings and most relevant findings obtained in this study are depicted in Figure 7. Stimulating OECs derived from primary cultures or human cell lines with these bacteria did not have a major impact in the production of cytokines such as IL-1β, TNFα, and IL-8, which are involved in the initiation of an inflammatory response. Likewise, such a bacteria cocktail did not result in MHC II expression by OECs, although this was readily induced by IFNγ. Co-stimulatory molecules (CD80/CD86) could not be induced by any assayed stimuli, but OECs expressed significant amounts of constitutive CD40. In contrast, others have described an increased CD80 and CD86 expression in an immortalized oral epithelial cell line, HOK-18A, along with MHC II expression in response to pathogenic bacteria (20). Expression of CD80/CD86 has also been described in intestinal epithelial cells (IEC) under inflammatory stimulation (34). Moreover, IECs isolated from patients with inflammatory bowel disease (IBD) expressed MHC II, CD80 and CD86 and were found capable of inducing CD4 T cell proliferation and IFNγ secretion (35). Thereby we cannot discard that other stimuli could promote the expression of CD80 and CD86 in OECs. In any case, given the expression of CD40 and MHC II, our results indicate that although these OECs might not activate naive CD4 T cells they are clearly ready to crosstalk with effector CD4 T cells and, according to the absence of CD80/CD86, perhaps limit their activity (36).

OECs share location and are in close contact with DCs within the oral mucosa. DCs act as sentinel cells, continuously sampling antigens, and microorganisms to present to T cells, thus bridging innate and adaptive immunity. As expected, DCs treated with the assayed bacteria cocktail reached a mature phenotype characterized by a high expression of CD86, CD80, CD40 and MHC II and a release of pro-inflammatory cytokines such IL-12 and TNFα (21). DCs cultivated in the presence of OECs displayed some features of maturation, judging by the expression of the indicated makers, which were not enhanced by the presence of bacteria. Moreover, the production of inflammatory cytokines (TNFα and IL-12) appeared decreased in DCs co-cultured with OECs even in the presence of bacteria. Overall, these results suggest that OECs modulate the maturation of DCs, making them refractory to bacterial stimulation and holding them in a semi-maturation state. Similar results have been described using Caco-2, a colonic epithelial cell line (8). DCs conditioned by supernatants from Caco-2 cells treated with bacteria exhibit a semi-mature phenotype and did not produce IL-12 (8). Likewise, renal tubular epithelial cells (ECs) and airway ECs induced a semi-mature phenotype on DCs, which could not be reverted by bacterial stimulation (37–39).

Mature DCs are responsible for activating and initiating effector T cell responses against foreign antigens, while semi-mature DCs have been involved in promoting tolerogenic T cell responses (40). Thereby, we investigated the response and phenotype of allogenic naive CD4 T cells activated with DCs in co-culture with OECs, with or without bacterial exposure. While DCs stimulated with bacteria pushed CD4 T cells to produce high levels of IFNγ and TNFα, consistent with a previous study (21), when OECs were present in the culture a total abrogation of the IFNγ released by CD4 T cells with a concomitant reduction of TNFα was noted. Interestingly, similar results were obtained with OECs in experiments in which naive CD4 T cells were activated using CD3/CD28 signaling in the absence of DCs; therefore ruling out any effect due exclusively to DCs. Abrogation of IFNγ and TNFα production was only observed when total CD4 T cells or Th1 polarized cells, were co-cultured with OECs and not with their conditioned media. This suggests a requirement for a direct or a very close contact between OECs and activated T cells to achieve that suppression. CD4 T cells stimulated with anti-CD3/CD28 antibodies were viable in co-cultures with OECs irrespective of the presence of inactivated bacteria, but they did barely express activation markers such as CD25 or CD69 and did not proliferate. This kind of inhibition of T cell responses has also been reported using other epithelial cells. Human biliary epithelial cells and colonic epithelial cells inhibited T cell responses without inducing T cell apoptosis (15, 16). Another study demonstrated that renal tubular epithelial cells presented immunosuppressive properties, being able of inhibiting CD4 and CD8 T cell activity, but, in contrast to our findings, inducing T cell apoptosis in a contact-dependent manner (17). Lung epithelial cells have also been reported to inhibit DC-induced T cell proliferation and cytokine release. This effect was predominantly contact-dependent and partly mediated by TGFβ (41). In contrast, colonic epithelial cells have been shown to prevent CD4 T cell activation by professional APCs in a contact-dependent manner but independent of TGFβ (15). Likewise, TGFβ did not seem to mediate CD4 T cell inhibition by H413 OECs, as IFNγ and TNFα production did not increase by adding TGFβ blocking antibodies in the culture. The possibility that OECs were consuming the produced IFNγ and TNFα was also unlikely, even though they have been described to express receptors for these cytokines (42, 43), as only a minor amount of the IFNγ and TNFα reduction could be explained by this fact.

CD4 T cells recovered from OECs co-cultures were hyporesponsive to re-stimulation with anti-CD3/CD28 antibodies as they were able to produce only low amounts of IFNγ and TNFα. A similar effect has been observed for CD4 T cells in contact with retinal pigmented epithelial cells, which remained unresponsive to new antigenic stimulation (44). In contrast, T cells recovered from colonic epithelial cell or renal tubular epithelial cell co-cultures were able to respond to a further antigenic stimulation (15, 17). Thus, OECs may be promoting a functional inactivation of T cells similar to anergy (45). Anergy is one of the main mechanisms involved in oral tolerance, especially under high antigen concentration, conditions in which Treg induction is poor (46). T cell anergy can be induced by altered antigenic presentation, by antigen recognition in absence of costimulation or by colligation of inhibitory molecules such as PD-1 or CTLA-4 (47–51). However, anergy is mainly induced in naive T cells while we used total CD4 T cells. Hence, we cannot discard some other phenomena including T cell exhaustion (52). Thus, the precise mechanism by which OECs rendered T cells unresponsive remains to be elucidated but it seems to be cell-cell contact dependent and likely facilitated by MHC II and CD40 expression on OECs.

## Conclusion

Our results indicate that OECs downregulate some DC functions and suppress local T cell responses in a rather constitutive manner. This behavior likely evolved to avoid harmful immune responses against residing bacteria and can favor oral treatments aimed to induce immune tolerance, but it needs to be overcome for treatments aimed to enhance or induce immunological protective memory.

Protection from undesired and/or excessive immune responses is of paramount relevance for survival and organisms must have evolved with various mechanisms to achieve it. Currently, those involving the active induction and harness of immune cells with tolerogenic phenotype has focused most of the researcher's attention (53) However, while these mechanisms are crucial and contribute to control immune responses, our results indicate that, overall, they are shadowed by the inherent ability of epithelial cells to restrain uncontrolled immune cell responses.

## Data Availability

All datasets generated for this study are included in the manuscript and/or the Supplementary Files.

## Author Contributions

EL and PR: conceptualization. MM-A, JS-T, MG-P, AT-G, and JS: methodology. MM-A, JS-T, JS, EL, and PR: investigation. JS-T, MG-P, and EL: writing-original draft. EL, JS, and PR: final writing and editing.

### Conflict of Interest Statement

JS was employed by company Inmunotek. The remaining authors declare that the research was conducted in the absence of any commercial or financial relationships that could be construed as a potential conflict of interest.

## Supplementary Material

Figure S1 OEC instruction alters cytokine profiles in CD3/CD28-activated naive CD4 T cells. We examined the levels of IFNγ, TNFα, IL-2, IL-4, TGFβ and IL-10 by ELISA on cultures consisting of CD3/CD28-activated naive CD4 T cells alone (CD4 Ctrl), in the presence of OEC (CD4:OEC) or their conditioned media (CD4:CM). The experiments were carried out using H413, TR146 cell lines and primary OECs. Media and MV130 conditions are represented by gray and black bars, respectively. Data were obtained from a total of four independent experiments and we plotted mean values with error bars corresponding to SEM. Statistically significant differences (*p* < 0.05) between the different CD4 T cells conditions and CD4 T cells Ctrl were noted as “^#^” for media or as “^*^” for MV130.
