# Supplementary figures and images for: Human Oral Epithelial Cells Impair Bacteria-Mediated Maturation of Dendritic Cells and Render T Cells Unresponsive to Stimulation
Source: Front Immunol. 2019 Jun 28;10:1434. doi: 10.3389/fimmu.2019.01434 (PMC6611079; doi:10.3389/fimmu.2019.01434)

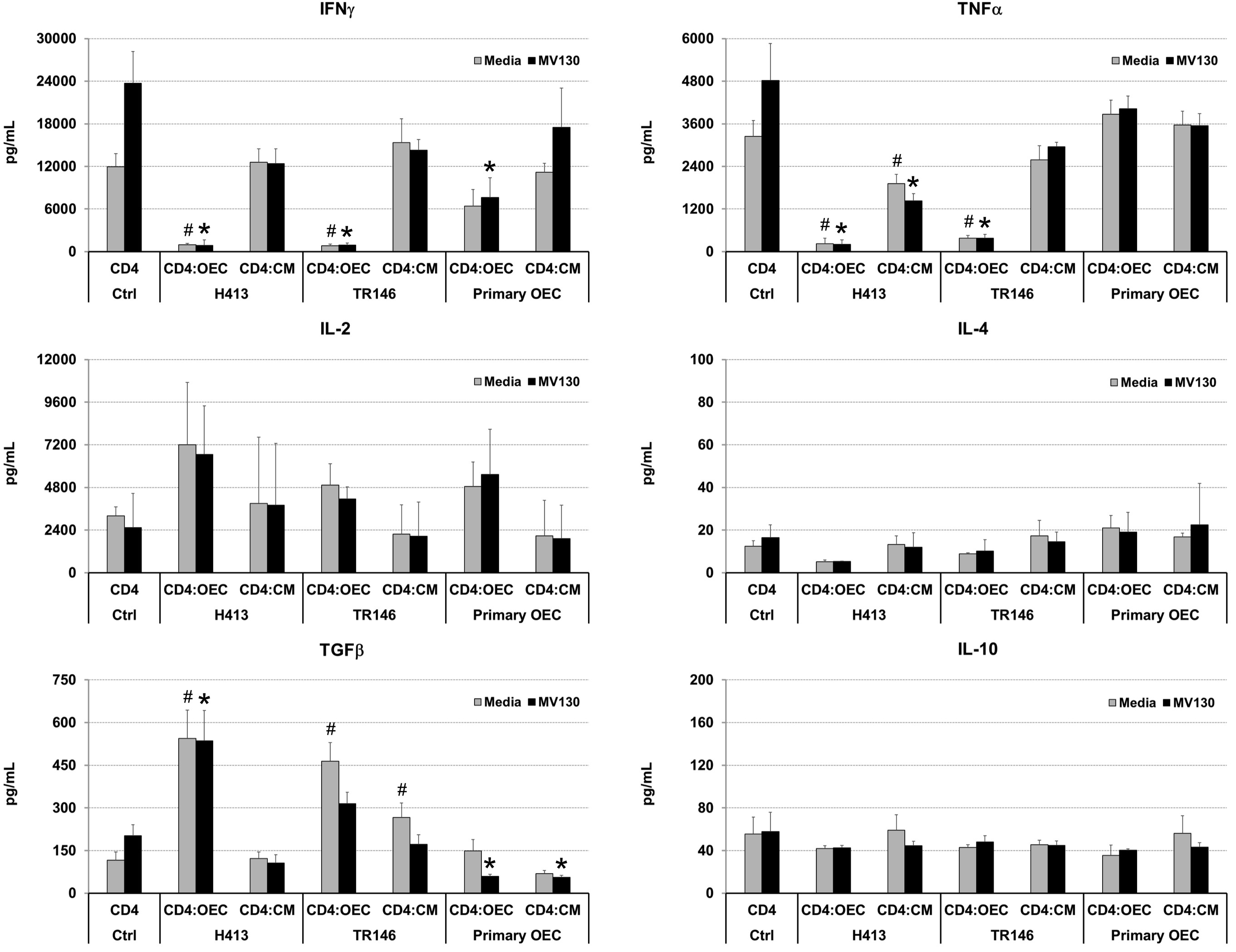

Supplement: Figure S1 — OEC instruction alters cytokine profiles in CD3/CD28-activated naive CD4 T cells. We examined the levels of IFNγ, TNFα, IL-2, IL-4, TGFβ and IL-10 by ELISA on cultures consisting of CD3/CD28-activated naive CD4 T cells alone (CD4 Ctrl), in the presence of OEC (CD4:OEC) or their conditioned media (CD4:CM). The experiments were carried out using H413, TR146 cell lines and primary OECs. Media and MV130 conditions are represented by gray and black bars, respectively. Data were obtained from a total of four independent experiments and we plotted mean values with error bars corresponding to SEM. Statistically significant differences (p < 0.05) between the different CD4 T cells conditions and CD4 T cells Ctrl were noted as “#” for media or as “*” for MV130. [file Image_1.jpg]
